# 尼妥珠单抗对不同化疗药物在肺癌PC9细胞中敏感性的影响及其机制

**DOI:** 10.3779/j.issn.1009-3419.2015.02.09

**Published:** 2015-02-20

**Authors:** 宇 肖, 宝山 曹, 莉 梁

**Affiliations:** 100191 北京，北京大学第三医院肿瘤化疗与放射病科 Department of Medical Oncology and Radiation Sickness, Peking University Third Hospital, Beijing 100191, China

**Keywords:** 尼妥珠单抗, 紫杉醇, 肺肿瘤, PC9细胞, Nimotuzumab, Paclitaxel, Lung neoplasms, PC9

## Abstract

**背景与目的:**

尼妥珠单抗是一种针对表皮生长因子受体的人源化IgG1型单克隆抗体，可在多种肿瘤中提高化疗和放射治疗的敏感性; 而非小细胞肺癌常用化疗药物有多种，尼妥珠单抗对这些药物敏感性的影响尚缺乏报道。本研究探讨尼妥珠单抗对顺铂、吉西他滨、紫杉醇、培美曲塞、长春瑞滨在非小细肺癌细胞中敏感性的影响及可能机制。

**方法:**

选择PC9人肺癌细胞作为观察对象，WST-1法检测细胞增殖率、流式细胞仪检测细胞周期、TUNEL法检测细胞凋亡率、免疫荧光染色联合激光扫描共聚焦显微镜下观察细胞内微管、微丝的分布情况。

**结果:**

尼妥珠单抗联合紫杉醇对PC9细胞的增殖抑制最为显著（*P* < 0.05），细胞凋亡率显著增加（*P*=0.013），细胞发生G_2_/M期阻滞的比例显著提高（*P* < 0.05）; 共聚焦显微镜下观察提示尼妥珠单抗促进PC9细胞中的微管和微丝聚集且排列整齐。

**结论:**

尼妥珠单抗能够显著提高紫杉醇在PC9细胞中的敏感性，机制可能与促进微管、微丝聚集和诱导G_2_/M期阻滞有关。尼妥珠单抗联合紫杉类药物可能是治疗非小细胞肺癌的一个潜在有效方案。

## Abstract (en)

**Background and objective:**

Nimotuzumab is a humanized IgG1 type monoclonal antibody targeting epidermal growth factor receptor, and can enhance chemosensitivity and radiosensitivity of certain cancers. The aim of this study is to investigate the effects of nimotuzumab on the chemosensitivities of PC9 human lung adenocarcinoma cells to common chemtherapeutic drugs including ciaplatin, gemcitabine, paclitaxel, pemetrexed and vinorelbine, and to elucidate possible mechanisms.

**Methods:**

PC9 human lung adenocarcinoma cell line was used in the study. Cell proliferation was determined by WST-1 assay and cell apoptosis was detected by TUNEL assay. Cell cycle distribution was analyzed by DNA analysis with FACS. Tublin and microfilaments were observed by immunofluorescence staining.

**Results:**

Nimotuzumab significantly enhanced the chemosensitivity of PC9 cells to paclitaxel. Cell proliferation was inhibited significantly (*P* < 0.05) and cell apoptosis rate was higher in nimotuzumab combined with low dose paclitaxel (0.05 μg/mL) group (*P*=0.013). G_2_/M arrest was increased significantly by nimotuzumab combined with paclitaxel group (*P* < 0.05). Nimotuzumab caused aggregation of tublin and microfilaments into well organized microtubules.

**Conclusion:**

Nimotuzumab enhanced the chemosensitivity of PC9 cell to paclitaxel by enhancing G_2_/M arrest and aggregation of tublin and microfilaments. Therefore, Nimotuzumab combined with taxane drugs could be a potential effective regimen in non-small cell lung cancer.

肺癌目前是全球发病率第一位的恶性肿瘤，非小细胞肺癌（non-small cell lung cancer, NSCLC）约占肺癌的80%^[1]^，确诊时约75%的NSCLC患者处于进展期，大多数进展期NSCLC患者5年生存率不足5%^[2]^。过去20年里，随着化疗新药（如紫杉类药物、培美曲塞、吉西他滨等）、针对表皮生长因子受体（epidermal growth factor receptor, *EGFR*）基因突变的靶向药物（如吉非替尼、厄洛替尼、埃克替尼和阿法替尼等）以及针对间变淋巴瘤激酶（anaplastic lymphoma kinase, *ALK*）基因易位的靶向药物（如克唑替尼）的出现，进展期肺癌患者的生存得到了明显改善，患者中位无进展生存期达到8个月-12个月^[3-9]^，但仍不能治愈。因此，寻找新的药物和新的治疗手段非常必要。

尼妥珠单抗是一针对EGFR人源化的IgG1型单克隆抗体，在多种肿瘤中其可以提高化疗和放疗的敏感性，并可逆转耐药^[10-15]^。在与放疗联合时，尼妥珠单抗通过使肿瘤细胞发生G_2_/M期阻滞，进而提高肿瘤的放射敏感性^[13]^。目前NSCLC常用化疗药物包括培美曲塞、吉西他滨、紫杉类、长春碱类、铂类药物多种，这些药物作用于肿瘤细胞的周期并不相同，尼妥珠单抗对这些药物的敏感性尚缺乏报道。因此，探讨尼妥珠单抗与这些药物联合对NSCLC的影响及其机制，对于优化治疗方案、提高疗效、减轻毒性和降低患者经济负担尤为重要。本研究通过体外实验，以PC9细胞为观察对象，探讨尼妥珠单抗同NSCLC中常用化疗药物联合对PC9细胞的细胞增殖、细胞凋亡、细胞周期的影响，并分析可能机制，为临床合理用药提供理论依据。

## 1 材料和方法

### 1.1 材料和试剂

PC9细胞株来自北京肿瘤医院基础实验室; RPMI-1640培养基、胎牛血清（FBS）、磷酸盐缓冲液（PBS）、0.25%胰蛋白酶、无酚红HBSS缓冲盐均购于Gibco公司; 赫斯特荧光染料33342（Hochest 33342）、碘化吡啶（PI）、N-（2-羟乙基）哌嗪-N'-2-乙烷磺酸（HEPES）购于Sigma-aldrich^®^公司; 顺铂注射液（5 mg/mL）购于云南个旧生物药业有限公司，培美曲塞（100 mg/支）及吉西他滨（200 mg/支）购于美国礼来公司，紫杉醇（30 mg/支）购于美国施贵宝公司，长春瑞滨（10 mg/支）购于皮尔法伯公司; 兔抗-微管蛋白（Tublin）购于Abcam^®^公司; 罗丹明标记的鬼笔环肽（Phalloidin）购于Cytoskeleton^®^公司; FITC-抗兔二抗购于Santa Cruz^®^公司。WST-1增殖试剂盒购于Roche^®^公司（美国）。96孔板（Costar^®^公司）; 其他试剂均为国产分析纯。

### 1.2 实验方法

#### 1.2.1 细胞培养

PC9细胞培养在含有10%胎牛血清的RPMI-1640细胞培养基中，37 ℃、5%CO_2_培养箱中培养。细胞长满瓶底80%-90%时用0.25%胰蛋白酶消化传代，取对数生长期的细胞进行实验。

#### 1.2.2 药敏试验

① 5×10^3^，12 h后加药。分成空白对照、顺铂、吉西他滨、培美曲塞、紫杉醇、长春瑞滨6组。化疗药物浓度依次为：0.01 μg/mL、0.1 μg/mL、1 μg/mL、10 μg/mL、100 μg/mL、1, 000 μg/mL。加药24 h后利用WST-1增殖检测试剂盒按照说明进行检测，分别计算药物的半数抑制浓度（50% inhibitory concentration, IC_50_）值。②根据药物的IC_50_值，每种药物选低于IC_50_值的1个浓度，分别设化疗组和化疗药+尼妥珠单抗联合组，尼妥珠单抗浓度设定为100 μg/mL。应用WST-1增殖检测试剂盒进行检测细胞增殖率，比较单药组和联合组对细胞抑制程度。细胞增殖率=（实验组平均OD值/对照组平均OD值）×100%。实验重复3次。

#### 1.2.3 细胞周期检测

1×10^6^ 12 h后分成PBS、顺铂2.5 μg/mL、顺铂2.5 μg/mL+妥珠单抗100 μg/mL，紫杉醇0.05 μg/mL，紫杉醇0.05 μg/mL+尼妥珠单抗100 μg/mL及紫杉醇5 μg/mL 6组，24 h后收集细胞，PBS洗涤2次，将细胞重悬于PBS中，加入70%乙醇固定，4 ℃孵育过夜。离心去除固定液，将细胞重悬于含有RNase A（250 mg/mL）的PBS中，加入碘化吡啶（50 mg/mL）37 ℃孵育30 min。应用FACSCalibur流式细胞仪（BD Biosciences）检测细胞DNA含量分布。实验重复3次。

#### 1.2.4 细胞凋亡检测

凋亡检测的细胞处理同细胞周期检测，收集的PC9细胞按照TUNEL凋亡检测试剂盒（Roche, Bioscience, Inc, 美国）说明进行处理，通过流式细胞仪（Becton Dickinson, San Jose, CA）检测细胞凋亡。实验重复3次。

#### 1.2.5 免疫荧光染色

将PC9细胞培养在铺有盖玻片的24孔板内，12 h后分别加入PBS、尼妥珠单抗、紫杉醇3组，加药24 h后应用4%多聚甲醛固定5 min，给予pH6.0、0.01 mol/L枸橼酸钠缓冲液进行抗原修复，应用1%BSA进行封闭，分别加入一抗：兔单抗-微管蛋白（Tublin，Abcam，ab179513，稀释比1:100）后加入抗兔的异硫氰酸荧光黄标记的羊抗兔IgG，洗涤后，加入罗丹明标记的鬼笔环肽（Phalloidin，稀释比1:200）; 应用DAPI染细胞核。通过Leica SP5激光扫描共聚焦显微镜观察各组荧光染色情况。

### 1.3 统计学方法

采用SPSS 11.0软件进行统计分析。计量资料采用*t*检验，计数资料采用卡方检验，采用Probit分析计算药物的IC_50_值。以*P* < 0.05为差异有统计学意义。

## 2 结果

### 2.1 不同化疗药物单用对PC9细胞增殖的影响

顺铂、紫杉醇、吉西他滨、培美曲塞和长春瑞滨在所检测的浓度和时间范围内对PC9细胞增殖的影响差别显著（图 1）。Probit分析法计算各种药物在PC9细胞中的IC_50_值，药物的IC_50_依次为：顺铂0.900 μg/mL、紫杉醇0.136 μg/mL、吉西他滨54.387 μg/mL、培美曲塞56.253 μg/mL和长春瑞滨2.036 μg/mL。

**1:**
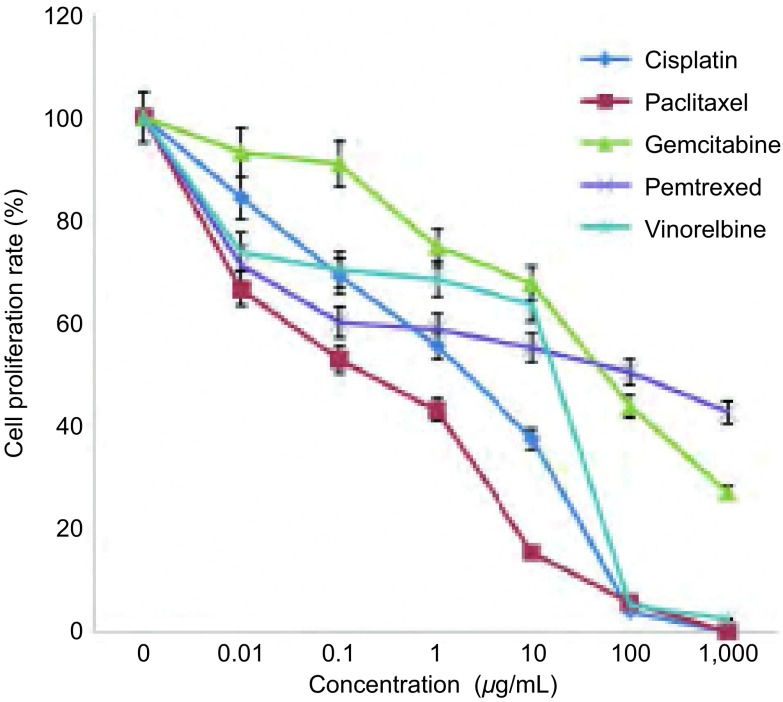
不同化疗药物对PC9细胞增殖影响（24 h） Effects of different chemotherapeutic drugs on PC9 cell proliferation after treatment (24 h)

#### 2.2 不同化疗药物联合尼妥珠单抗对细胞增殖的影响

本研究根据文献^[14, 16]^回顾，尼妥珠单抗的浓度设定为100 μg/mL，为了更好地体现联合组药物对细胞增殖的影响，本研究中化疗药物浓度选择均低于对应的IC_50_。紫杉醇、顺铂、吉西他滨、培美曲塞和长春瑞滨的浓度依次为0.05 μg/mL、0.5 μg/mL、2.5 μg/mL、2.5 μg/mL、1 μg/mL。结果显示，联合组对PC9细胞增殖抑制强于单药组，其中尼妥珠单抗联合紫杉醇对PC9细胞增殖抑制最为显著（*P* < 0.05）（图 2）。

**2:**
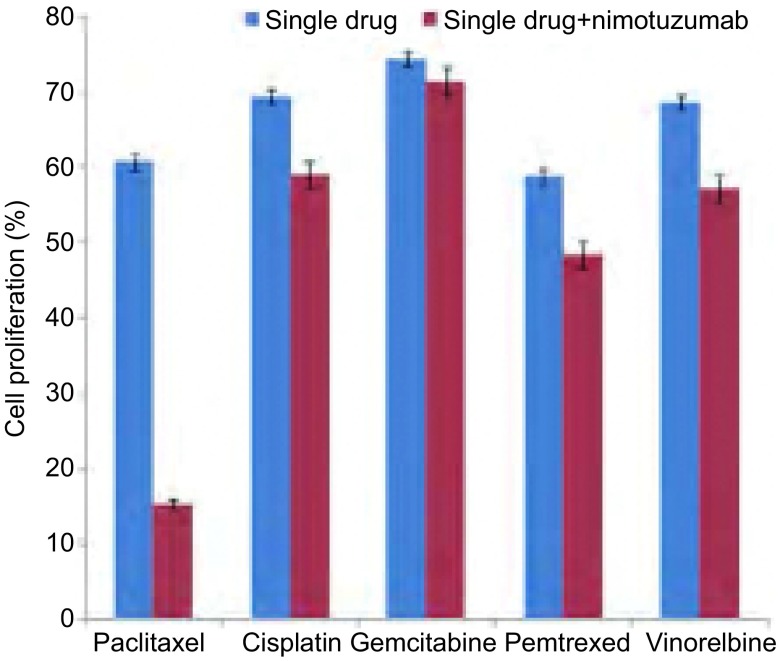
化疗药物±尼妥珠单抗（100 μg/mL）对PC9细胞增殖的影响 Effects of chemotherapeutic drugs with or without nimotuzumab (100 μg/mL) on PC9 cell proliferation

#### 2.3 PC9细胞周期分布情况

本研究根据细胞增殖抑制结果和紫杉醇作用于G_2_/M期特点，特选择紫杉醇和顺铂（非周期特异性药物）为代表，分析了化疗单药组和化疗联合尼妥珠单抗组对PC9细胞周期的影响。结果发现尼妥珠单抗联合紫杉醇（0.05 μg/mL）组PC9细胞G_2_/M期阻滞细胞比例67.8%±2.3%显著高于单药紫杉醇组26.3%±3.1%（*P* < 0.05）。尼妥珠单抗联合低剂量（0.05 μg/mL）紫杉醇导致的G_2_/M期细胞阻滞比例也显著高于紫杉醇大剂量（5 μg/mL）单药组; 尼妥珠单抗联合顺铂对PC9细胞的周期分布无明显影响（*P* > 0.05）（图 3）。

**3:**
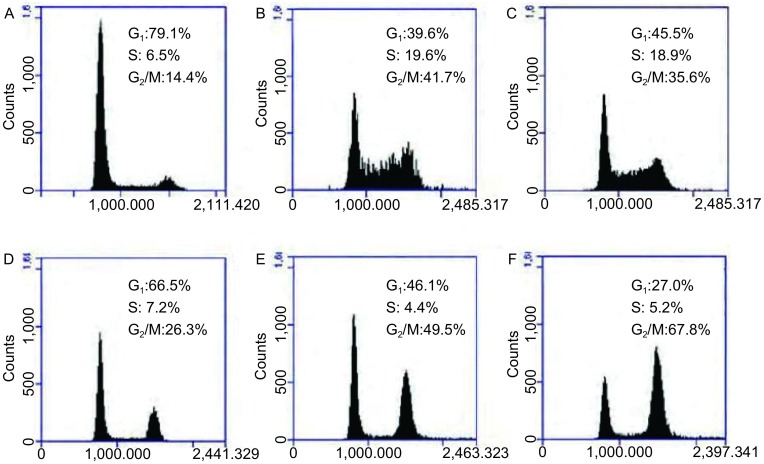
PC9细胞周期分布情况。A：空白对照；B：顺铂（2.5 μg/ mL）；C：顺铂（2.5 μg/mL）+尼妥珠单抗（100 μg/mL）；D：紫杉醇（0.05 μg/mL）；E：紫杉醇（5 μg/mL）；F：紫杉醇（0.05 μg/ mL）+尼妥珠单抗（100 μg/mL） Cell cycle distribution of PC cells in different groups. A: control; B: cisplatin (2.5 μg/mL); C: cisplatin (2.5 μg/mL)+nimotuzumab (100 μg/mL); D: paclitaxel (0.05 μg/mL); E: paclitaxel (5 μg/mL); F: paclitaxel (0.05 μg/mL)+nimotuzumab (100 μg/mL).

#### 2.4 PC9细胞凋亡情况

根据上述细胞增殖和周期分析结果，本研究选择了顺铂和紫杉醇做为代表性药物，观察化疗药物单独或联合使用尼妥珠单抗对细胞的凋亡影响。结果发现，化疗药物无论联合或不联合尼妥珠单抗组中，PC9细胞凋亡率均显著高于对照组（*P* < 0.05）; 尼妥珠单抗联合紫杉醇（0.05 μg/mL）组所致的PC9细胞凋亡率9.63%±2.04%显著高于紫杉醇单药组（0.05 μg/mL）中的4.25%±0.90%（*P*=0.013），且高于紫杉醇大剂量（5 μg/mL）单药组5.44%±1.00%;尼妥珠单抗联合顺铂组PC9细胞凋亡率1.43%±0.33%与顺铂单药组1.00%±0.23%无统计学差异（*P*=0.134）（图 4）。

**4:**
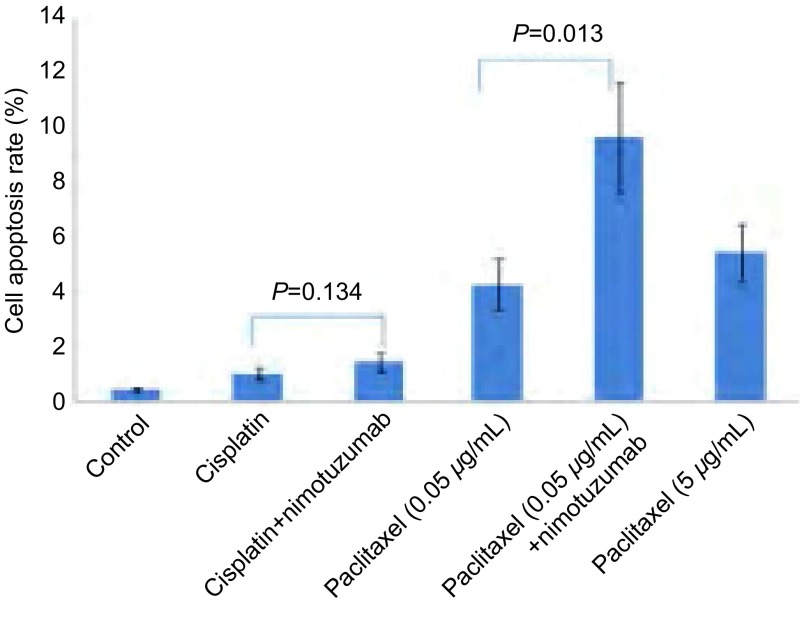
顺铂和紫杉醇±尼妥珠单抗对PC9细胞凋亡的影响 Effects of chemotherapeutic drugs (cisplatin, paclitaxel) with or without nimotuzumab (100 μg/mL) on PC9 cell apoptosis

#### 2.5 尼妥珠单抗与化疗对PC9细胞的微管、微丝影响

上述结果显示，尼妥珠单抗在与紫杉醇联合时使PC9细胞显著发生G_2_/M期阻滞，并提高了紫杉醇对肿瘤细胞的杀伤。而长春瑞滨药理学作用表明，其也主要是通过作用于肿瘤的G_2_/M期细胞发挥作用。紫杉醇和长春瑞滨二者均作用于G_2_/M期，但前者抑制微管解聚，后者促进微管解聚。因此，本研究应用免疫荧光染色，共聚焦显微镜下观察尼妥珠单抗对PC9细胞中微管和微丝分布的影响。结果发现尼妥珠单抗能够使PC9细胞中的微管和微丝聚集且排列更整齐，与紫杉醇促进微管整齐聚集排列相一致（图 5）。

**5:**
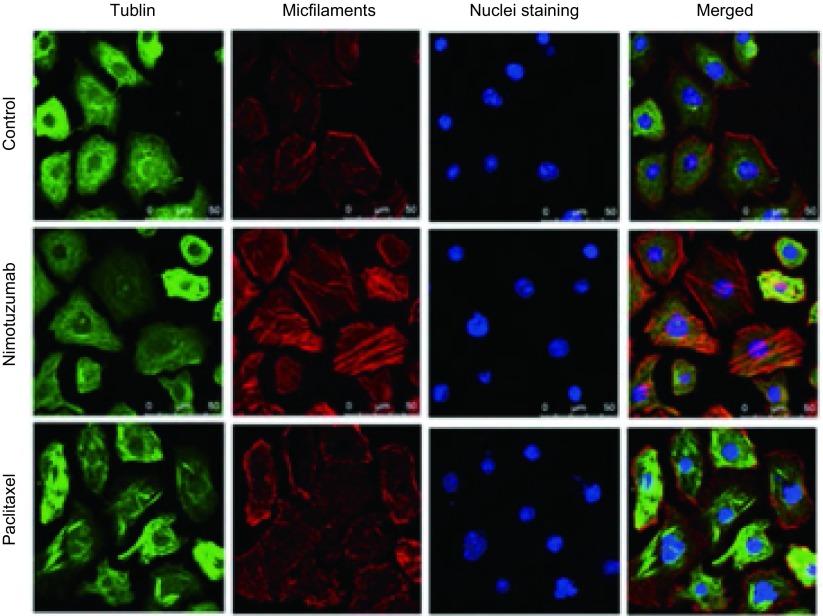
PC9细胞中微管、微丝分布情况（间接免疫荧光染色，×630） Tublin and microfilaments distribution of PC9 cells in different drug groups (indirect immunofluoresence staining, ×630)

## 3 讨论

EGFR信号通路激活在多种肿瘤的形成、生长和维持中发挥着重要作用，在NSCLC中也是如此^[3, 17]^。因此，EGFR信号阻断成为NSCLC潜在的治疗靶点。目前阻断EGFR通路的药物包括2大类，一种是针对*EGFR*基因突变的小分子酪氨酸激酶抑制剂，常用药物包括吉非替尼、厄洛替尼和埃克替尼^[3-8]^;另一种是针对EGFR的单克隆抗体，主要包括西妥昔单抗、帕尼单抗和尼妥珠单抗^[15, 17, 18]^。FLEX研究证实西妥昔单抗联合长春瑞滨及顺铂可以提高晚期NSCLC的客观缓解率以及延长患者生存^[17]^，西妥昔单抗可提高多西紫杉醇方案的疗效^[18]^。但随后研究中，西妥昔单抗并没有提高培美曲塞联合铂类药物在NSCLC中的疗效^[19]^。上述研究提示针对EGFR的单抗类药物对化疗疗效的影响或许与化疗药物的种类有关。

尼妥珠单抗是一针对EGFR的人源化IgG1型单克隆抗体，阻断EGF、TGF-α及其他配体与EGFR结合，同EGFR结合的亲和力中等，仅为西妥昔单抗的1/10^[20]^，或许这是尼妥珠单抗毒性远低于西妥昔单抗的原因。目前尼妥珠单抗在脑胶质瘤、胶质母细胞瘤、头颈部肿瘤、胰腺癌、食管癌等多种肿瘤的治疗中发挥了重要作用，可提高化疗、放疗疗效，并延长患者生存期^[12, 15, 21-25]^。近期在NSCLC临床研究中，尼妥珠单抗能够提高化疗药物的客观缓解率^[26]^，并对*EGFR*基因突变的患者也有效。

本研究选择的PC9细胞株是含有*EGFR*基因突变的NSCLC细胞株，顺铂、吉西他滨、培美曲塞、紫杉醇、长春瑞滨是NSCLC中常用的五种化疗药物。尼妥珠单抗采用的剂量100 μg/mL为其他研究所推荐剂量^[14]^。研究发现尼妥珠单抗对紫杉醇的增敏作用最明显。因对其他药物增敏效果不明显，为降低资源消耗，研究选择了周期非特异性药物顺铂和紫杉醇进行了细胞凋亡、细胞周期分布的比较。尼妥珠单抗联合紫杉醇提高了PC9细胞的凋亡率，使PC9细胞发生G_2_/M期阻滞的细胞比例增加，而对周期非特异性药物顺铂无影响。提示尼妥珠单抗联合紫杉醇时细胞增殖抑制明显的原因或许是尼妥珠单抗促进了紫杉类药物所作用的G_2_/M期细胞比例升高有关。这与Lin等^[13]^报道类似，即尼妥珠单抗通过提高A549细胞G_2_/M期阻滞提高放疗的敏感性。但Song等^[14]^研究发现尼妥珠单抗在逆转多西紫杉醇耐药的SPC-A1/DTX细胞株时，主要是通过G1期阻滞细胞比例增加而提高SPC-A1/DTX细胞株对多西紫杉醇的敏感性。Jiang等^[16]^在研究A549细胞时，也发现尼妥珠单抗主要是通过使A549细胞发生G_1_期阻滞，S期细胞减少，进而促进A549细胞凋亡。这些差别的产生或许与研究所用细胞株不同以及细胞前期处理不同有关。

对同属于G_2_/M期周期特异性药物长春瑞滨而言，尼妥珠单抗没有明显提高长春瑞滨对PC9细胞的增殖抑制。药理学研究表明长春瑞滨主要通过促进微管解聚发挥细胞毒作用，而紫杉醇是通过抑制微管解聚发挥细胞毒作用。本研究通过应用标记微管蛋白以及应用鬼笔环肽标记微丝，结果发现尼妥珠单抗可以促使细胞微管、微丝聚集更明显、排列更整齐，这与紫杉醇的作用机理类似。因此尼妥珠单抗或许是因使PC9细胞微管、微丝聚集，从而促进抑制微管解聚，诱导肿瘤细胞发生G_2_/M期阻滞，而发挥对紫杉类药物的药敏作用。近期Babu等^[26]^应用多西紫杉醇+卡铂±尼妥珠单抗一线治疗进展期NSCLC患者的多中心期研究，结果发现尼妥珠单抗联合紫杉类药物组同紫杉类单纯化疗组相比，其客观缓解率分别为：54% *vs* 34.5%（*P* < 0.05）。提示尼妥珠单抗联合紫杉类药物或许是进展期NSCLC一线治疗或者新辅助治疗方案的合理选择。

本研究存在一定局限性，细胞株选择范围小，进一步体外研究将扩大细胞株筛选范围，明确尼妥珠单抗对化疗药物增敏的影响; 此外，尼妥珠单抗在体内还可以通过抑制EGFR信号，阻断血管生成、肿瘤浸润等发挥作用，因此在体外研究的基础上，需要进行体内和临床研究验证。

总之，尼妥珠单抗对不同化疗药物在PC9细胞中的增敏效果存在差别，其通过使微管、微丝聚集且整齐排列，阻滞PC9细胞于G_2_/M期，从而明显提高紫杉醇敏感性。因此，尼妥珠单抗联合紫杉类药物方案或许是一线或二线治疗NSCLC理想选择。

## References

[1] Siegel R, Naishadham D, Jemal A (2013). Cancer statistics, 2013. CA Cancer J Clin.

[2] Goldstraw P, Crowley J, Chansky K (2007). The IASLC Lung Cancer Staging Project: proposals for the revision of the TNM stage groupings in the forthcoming (seventh) edition of the TNM Classification of malignant tumours. J Thorac Oncol.

[3] Maemondo M, Inoue A, Kobayashi K (2010). Gefitinib or chemotherapy for non-small-cell lung cancer with mutated EGFR. N Engl J Med.

[4] Mok TS, Wu YL, Thongprasert S (2009). Gefitinib or carboplatin-paclitaxel in pulmonary adenocarcinoma. N Engl J Med.

[5] Mitsudomi T, Morita S, Yatabe Y (2010). Gefitinib versus cisplatin plus docetaxel in patients with non-small-cell lung cancer harbouring mutations of the epidermal growth factor receptor (WJTOG3405): an open label, randomised phase 3 trial. Lancet Oncol.

[6] Rosell R, Carcereny E, Gervais R (2012). Erlotinib versus standard chemotherapy as first-line treatment for European patients with advanced *EGFR* mutation-positive non-small-cell lung cancer (EURTAC): a multicentre, open-label, randomised phase 3 trial. Lancet Oncol.

[7] Zhou C, Wu YL, Chen G (2011). Erlotinib versus chemotherapy as first-line treatment for patients with advanced *EGFR* mutation-positive non-small-cell lung cancer (OPTIMAL, CTONG-0802): a multicentre, open-label, randomised, phase 3 study. Lancet Oncol.

[8] Wu YL, Zhou C, Hu CP (2014). Afatinib versus cisplatin plus gemcitabine for first-line treatment of Asian patients with advanced non-small-cell lung cancer harbouring *EGFR* mutations (LUX-Lung 6): an open-label, randomised phase 3 trial. Lancet Oncol.

[9] Shaw AT, Kim DW, Nakagawa K (2013). Crizotinib versus chemotherapy in advanced ALK-positive lung cancer. N Engl J Med.

[10] Song H, Pan B, Yi J (2014). Featured article: autophagic activation with nimotuzumab enhanced chemosensitivity and radiosensitivity of esophageal squamous cell carcinoma. Exp Biol Med (Maywood).

[11] Qu YY, Hu SL, Xu XY (2013). Nimotuzumab enhances the radiosensitivity of cancer cells *in vitro* by inhibiting radiation-induced DNA damage repair. PLoS One.

[12] Solomon MT, Miranda N, Jorrin E (2014). Nimotuzumab in combination with radiotherapy in high grade glioma patients: a single institution experience. Cancer Biol Ther.

[13] Lin S, Yan Y, Liu Y (2015). Sensitisation of human lung adenocarcinoma A549 cells to radiotherapy by Nimotuzumab is associated with enhanced apoptosis and cell cycle arrest in the G_2_/M phase. Cell Biol Int.

[14] Song HZ, Yi J, Chen J (2012). Nimotuzumab increases chemosensitivity of human lung adenocarcinoma cell lines to docetaxel. Oncol Res.

[15] Su D, Jiao SC, Wang LJ (2014). Efficacy of nimotuzumab plus gemcitabine usage as first-line treatment in patients with advanced pancreatic cancer. Tumour Biol.

[16] Jiang YQ, Zhou ZX, Ji YL (2014). Suppression of EGFR-STAT3 signaling inhibits tumorigenesis in a lung cancer cell line. Int J Clin Exp Med.

[17] Pirker R, Pereira JR, Szczesna A (2009). Cetuximab plus chemotherapy in patients with advanced non-small-cell lung cancer (FLEX): an open-label randomised phase Ⅲ trial. Lancet.

[18] Fischer JR, Griesinger F, Fink T (2012). Docetaxel-carboplatin chemotherapy combined with cetuximab in patients with locally advanced or metastatic non small-cell lung cancer (NSCLC)-results of the nonrandomised phase Ⅱ study TaxErb. Lung Cancer.

[19] Kim ES, Neubauer M, Cohn A (2013). Docetaxel or pemetrexed with or without cetuximab in recurrent or progressive non-small-cell lung cancer after platinum-based therapy: a phase 3, open-label, randomised trial. Lancet Oncol.

[20] Boland W, Bebb G (2010). The emerging role of nimotuzumab in the treatment of non-small cell lung cancer. Biologics.

[21] Liang J E M, Wu G (2013). Nimotuzumab combined with radiotherapy for esophageal cancer: preliminary study of a phase Ⅱ clinical trial. Oncol Targets Ther.

[22] Rodriguez MO, Rivero TC, del Castillo Bahi R (2010). Nimotuzumab plus radiotherapy for unresectable squamous-cell carcinoma of the head and neck. Cancer Biol Ther.

[23] Reddy BK, Lokesh V, Vidyasagar MS (2014). Nimotuzumab provides survival benefit to patients with inoperable advanced squamous cell carcinoma of the head and neck: a randomized, open-label, phase Ⅱb, 5-year study in Indian patients. Oral Oncol.

[24] Basavaraj C, Sierra P, Shivu J (2010). Nimotuzumab with chemoradiation confers a survival advantage in treatment-naive head and neck tumors over expressing EGFR. Cancer Biol Ther.

[25] Wang Y, Pan L, Sheng XF (2014). Nimotuzumab, a humanized monoclonal antibody specific for the EGFR, in combination with temozolomide and radiation therapy for newly diagnosed glioblastoma multiforme: First results in Chinese patients. Asia Pac J Clin Oncol.

[26] Babu KG, Prabhash K, Vaid AK (2014). Nimotuzumab plus chemotherapy versus chemotherapy alone in advanced non-small-cell lung cancer: a multicenter, randomized, open-label phase Ⅱ study. Oncol Targets Ther.

